# Supplementary material for: Coxsackievirus and adenovirus receptor expression facilitates enteroviral infections to drive the development of pancreatic cancer
Source: Nat Commun. 2024 Dec 4;15:10547. doi: 10.1038/s41467-024-55043-x (PMC11615305; doi:10.1038/s41467-024-55043-x)
Supplement: Supplementary file 1 — Supplementary Information [file 41467_2024_55043_MOESM1_ESM.pdf]

Supplementary Figure S1

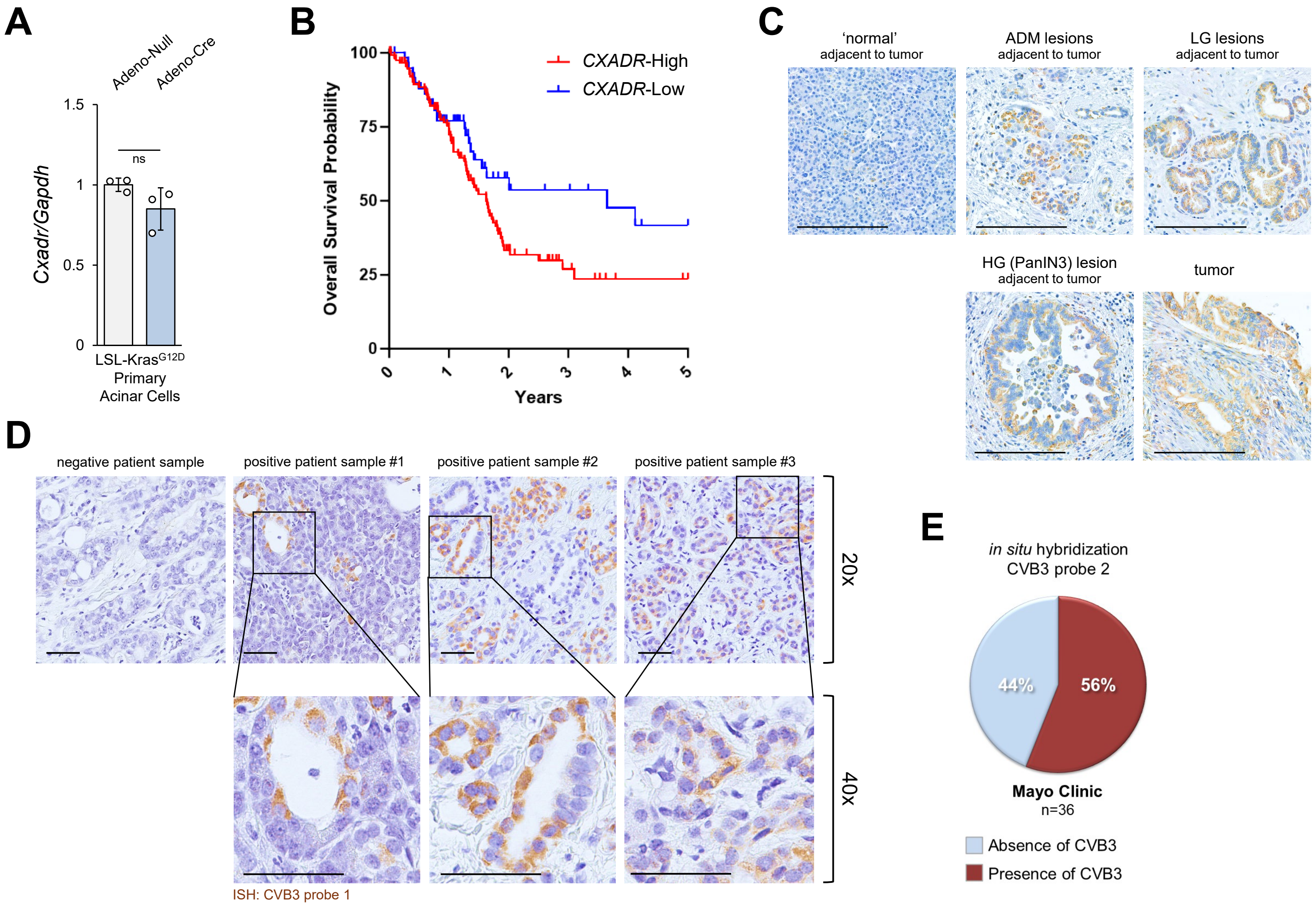

Supplementary Figure S1. A: *Cxadr*/CAR expression is not upregulated in Kras^{G12D}-expressing primary acinar cells. Primary acini were isolated from LSL-Kras^{G12D} mice and infected with Adeno-Null (control) or Adeno-Cre virus (to induce Kras^{G12D} expression). Cells were then cultured on plastic for 24 hours, harvested and subjected to quantitative RT-PCR analysis for *Cxadr*/CAR and *Gapdh*. The bar graph shows data from n=3 biological replicates. Data are presented as mean values -/+ SD. Source data are provided as a Source Data file. **B:** Overall survival for high (red; RSEM normalized count >=10.23; n=118) and low (blue; RSEM normalized count <10.23; n=60) *CXADR* expression in TCGA pancreatic adenocarcinoma primary tumor samples. Statistical significance determined with the Log-rank (Mantel-Cox) test (p=0.0442). **C: CAR expression in human normal acinar tissue and different lesion types adjacent to tumor.** IHC for CAR. Bar represents 200 µm. Shown are representative pictures from n=10 samples analyzed. **D: Addition to Fig. 2C.** Show is an example of a patient sample negative for CVB3 ISH and three different additional patient samples positive for CVB3 ISH. The bar indicates 50 µm. **E: Addition to Fig. 2D**. Serial sections of TMAs containing human PDA samples (Mayo Clinic n=36 samples) were subjected to ISH probe 2 for CVB3. The pie graph shows % presence or absence of CVB3 for the *in situ* hybridization data. Source data are provided as a Source Data file.

Supplementary Figure S2

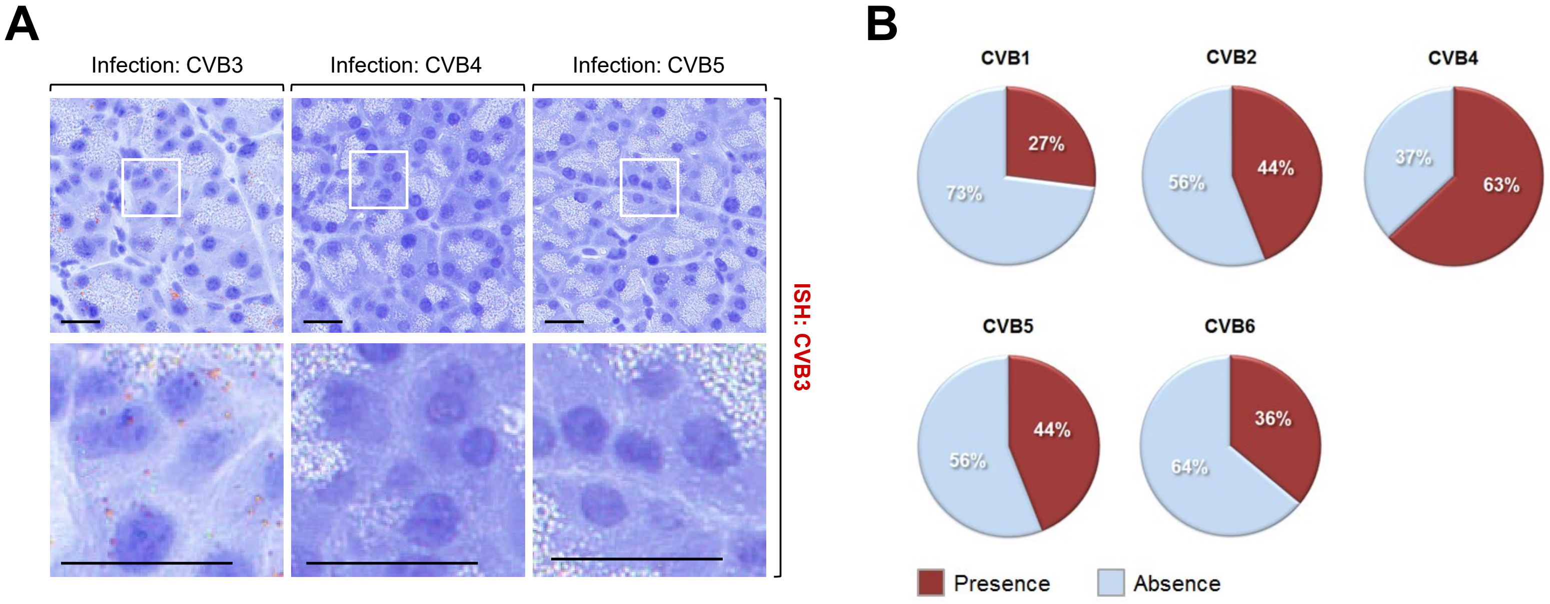

Supplementary Figure S2. A: Specificity of the CVB3 ISH probe2 used in Fig. 2D. FVB mice were infected with CVB3, CVB4 or CVB5 for 1-2 weeks. Pancreata were harvested and mice analyzed. Shown is an *in situ* hybridization (ISH) for CVB3 using ACD RNAscope 2.5 HD Brown kit and V-CVB3 (ACD #409291) as probe. This control experiment was conducted with 3 mice per treatment group. shown are representative pictures. The bar indicates 20 µm. **B:** Pancreatic cancer TMA slides (US Biomax, n = 60 patients) were analyzed for absence or presence of coxsackievirus CVB1, CVB2, CVB4, CVB5 and CVB6 using immunohistochemistry. Results are summarized in a pie graph. CVB1: 27.4 ± 3.1%, CVB2: 43.6± 9.7%, CVB4: 62.8 ± 9.2%, CVB5: 44 ± 11.6%, CVB6: 31.9 ± 3.6%. Source data are provided as a Source Data file.

Supplementary Figure S3

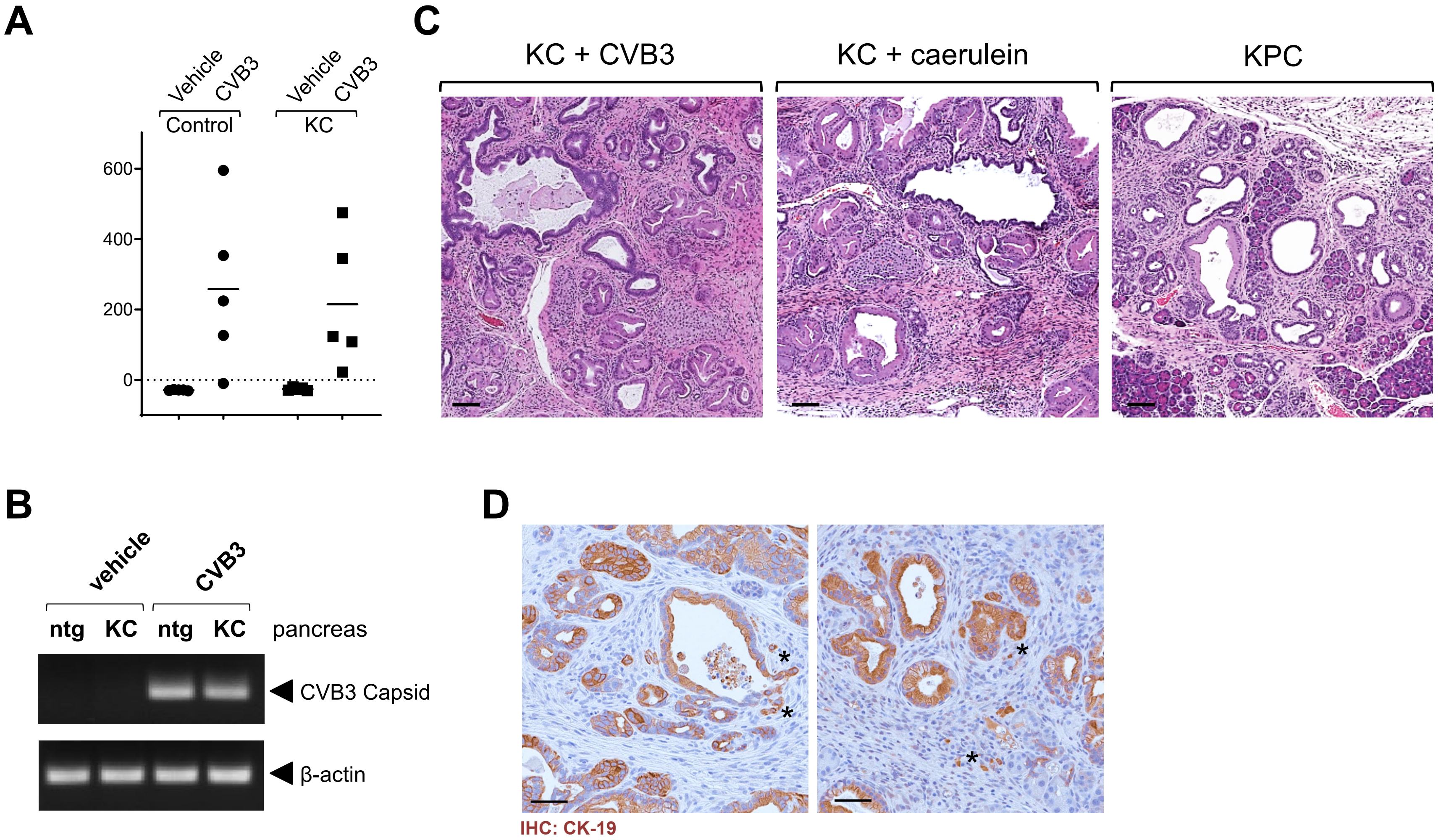

Supplementary Figure S3. A: Detection of antibodies directed against CVB3 in sera from n=5 ntg and n=5 KC mice, 6 weeks after infection with CVB3, using an ELISA assay. Source data are provided as a Source Data file. Bar reflects the mean. **B:** RT-PCR after RNA extraction (Trizol) from indicated pancreata from ntg and KC mice, 6 weeks after infection with CVB3. Primers: CVB3-Nancy capsid: 5'-CAACATGGGCACGCTATATG-3' and 5'-CTGGGTTGGAAGTTCACGTT-3' and β-actin: 5'-GGCCAGGTCATCACTATTGGCAAC-3' and 5'-CAGAGCAGTAATCTCCTTCTGCAT-3'. PCR conditions: 100 ng cDNA; Step 1: 94 °C, 3 min; Step 2: 94 °C ,15 sec; Step 3: 55 °C, 30 sec; Step 4: 68 °C, 3 min; Steps 2-4 repeat 55 cycles; Step 5: 68 °C, 10 min; Step 6: 4 °C. The samples derive from the same experiment but different gels for CVB3 Capsid and β-actin were processed in parallel. See Source Data for original gels. Source data are provided as a Source Data file. **C:** Representative pictures (from n=4 mice per condition) to compare pancreata of 14 weeks old KC mice that at an age of 8 weeks were one-time infected with CVB3, 18 weeks old KC mice that at an age of 8 weeks were treated with caerulein (75 μg/kg caerulein, 1 injection/hour over a period of 8 hours, 2 days in a row) to induce acute pancreatitis, and 15 weeks old KPC mice. Shown is an H&E staining. The bar is 100 μm. **D:** IHC for CK-19 indicating single cells of ductal origin in the stroma (asterisks). Representative pictures from n=3 mice analyzed.

Supplementary Figure S4

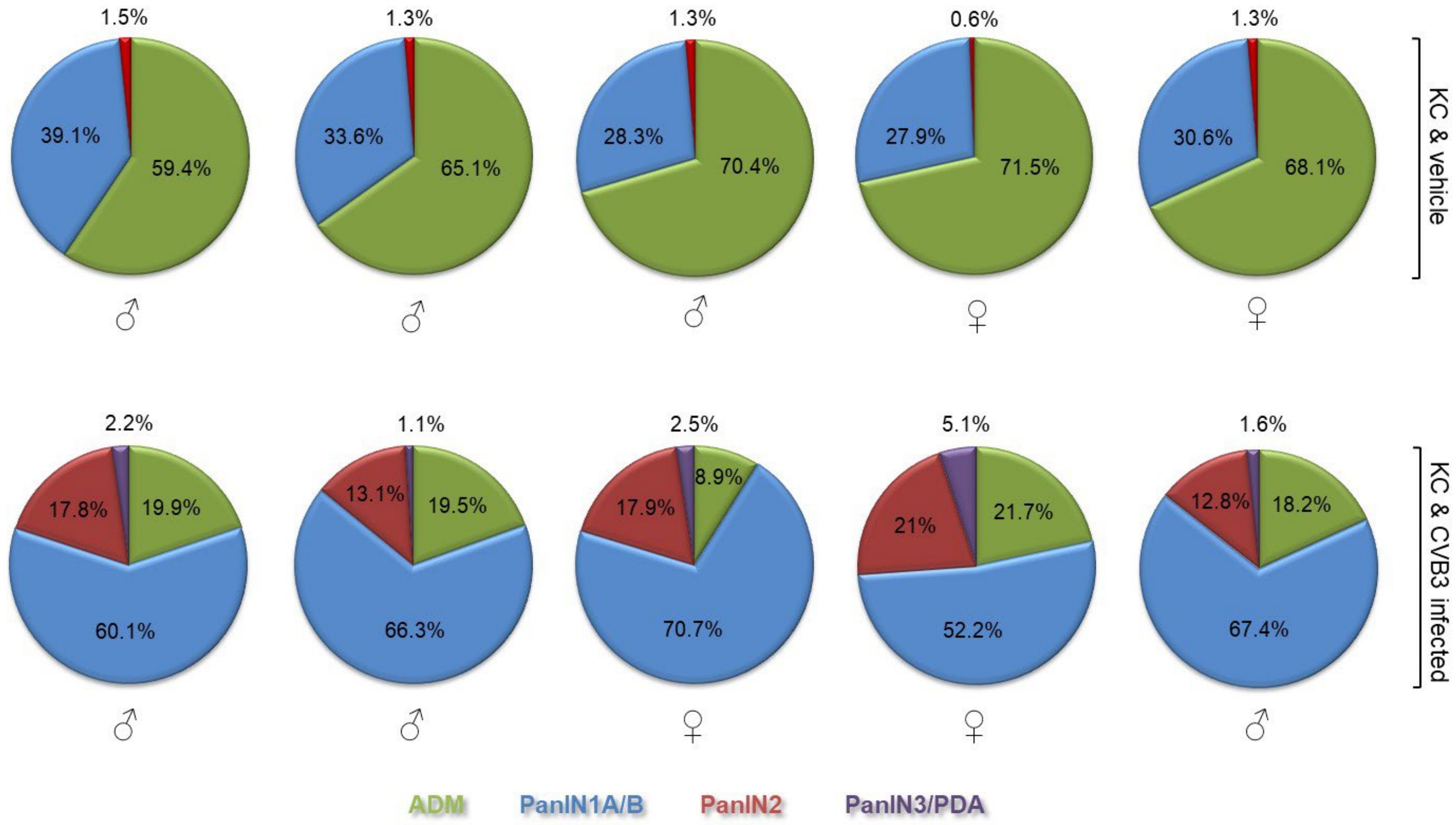

Supplementary Figure S4. Supplement to Figure 3D. Shown is the individual analyses of each mouse per experimental group (KC & vehicle *versus* KC & CVB3 infected) for ADM, PanIN1A/B, PanIN2 and PanIN3/PDA. The sex of each animal is indicated. Source data are provided as a Source Data file.

Supplementary Figure S5

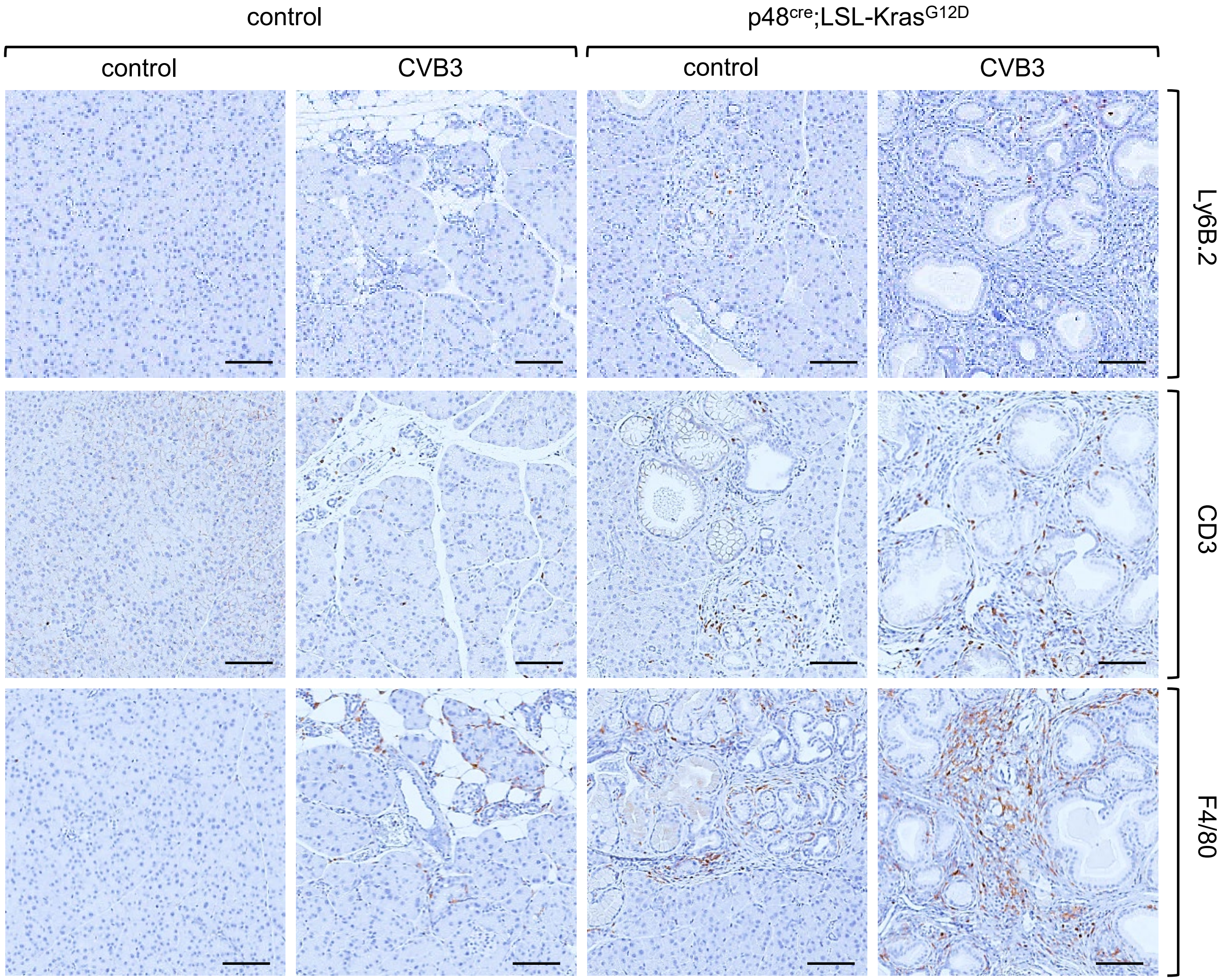

Supplementary Figure S5. Supplement to Figure 3F. Representative pictures (from n=5 samples each treatment group) for the quantification shown in Figure 3F. Pancreata were analyzed by IHC for presence of Ly6B.2 positive (neutrophils),CD3 positive (T-cells) or F4/80 positive (macrophages) cells. The bar indicates 100 µm.

Supplementary Figure S6

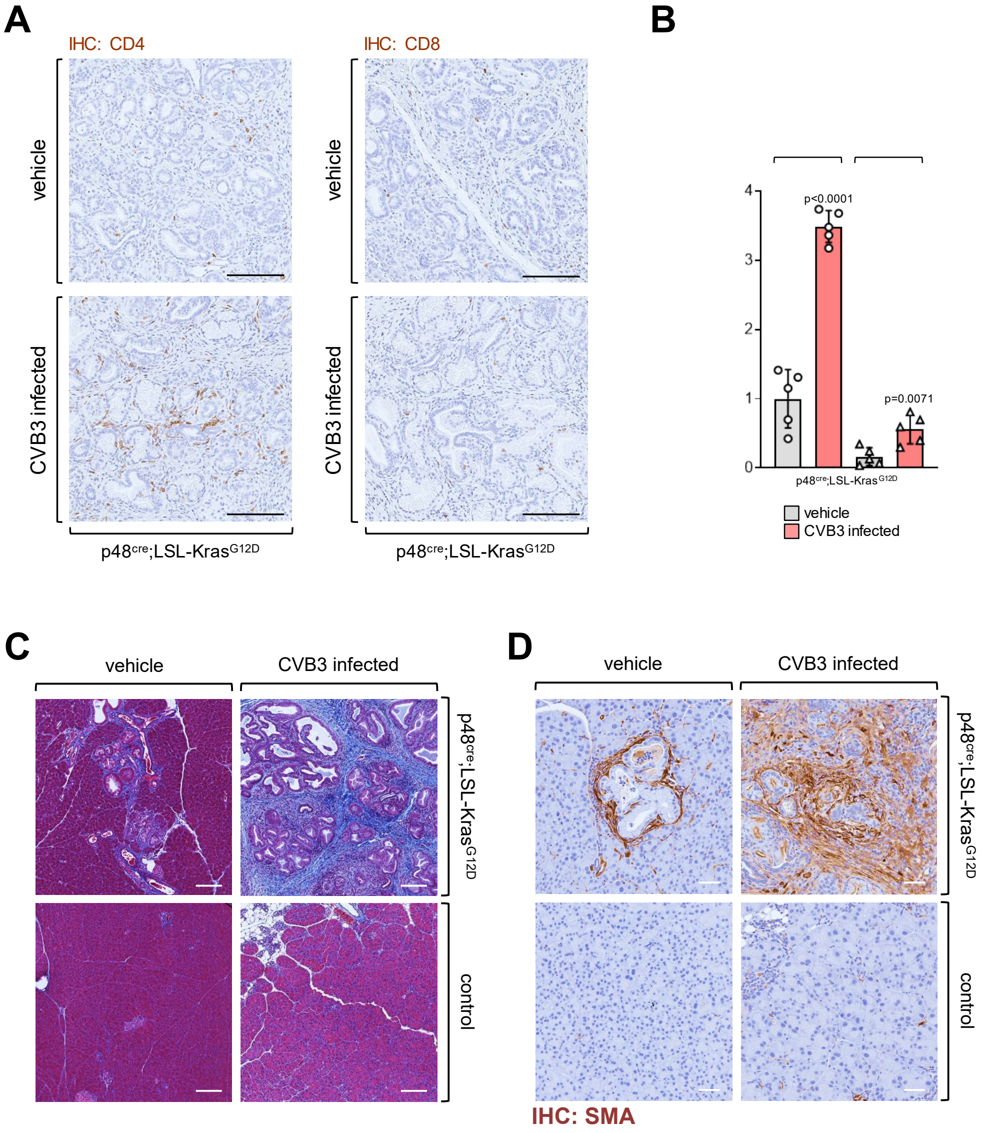

Supplementary Figure S6. A, B: CVB3 infection increases the presence of CD4+ T cells in pancreatic lesion areas. A: Pancreatic tissues of KC mice either vehicle treated or infected with CVB3 were analyzed by IHC for presence of CD4+ or CD8+ T cells. Shown is a representative lesion area corresponding to the analyses in S6B. The bar indicates 100 µm. **B:** Corresponding quantification of pancreata from n=5 mice per experimental group. Data are presented as mean values -/+ SD. Statistical significance between two sample conditions was determined using the two-tailed t-test and is indicated by a p value < 0.05. Source data are provided as a Source Data file. **C, D: CVB3 infected KC mice show increased fibrosis. C:** Pancreata of control or KC mice either vehicle treated or infected with CVB3 (n=5 per group) were stained with trichrome. Shown is a representative area. The bar indicates 200 µm. **D:** Pancreata of control or KC mice either vehicle treated or infected with CVB3 (n=5 per group) were analyzed by IHC for presence of smooth muscle actin (anti-SMA). Shown is a representative area. The bar indicates 50 µm.

Supplementary Figure S7

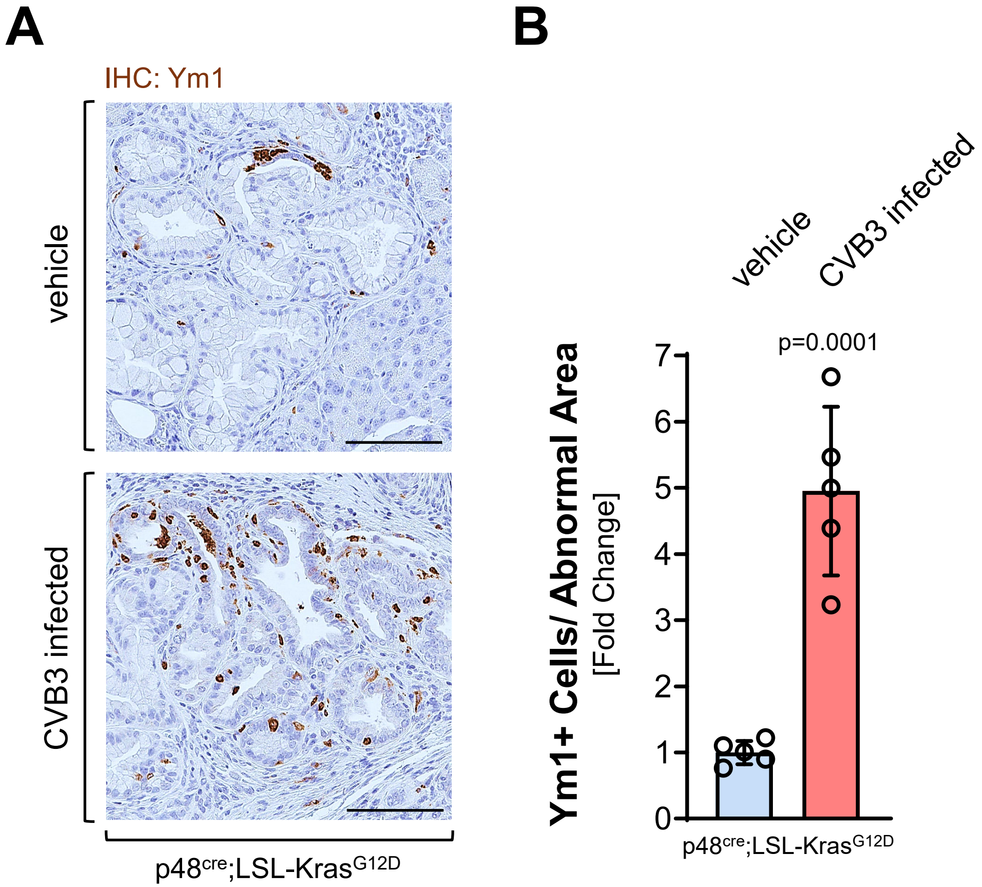

Supplementary Figure S7. CVB3 infection increases the expression of Ym1+ cells in pancreatic lesion areas. A: Pancreatic tissues of KC mice either vehicle treated or infected with CVB3 (n=5 per group) were analyzed by IHC for presence of Ym1+ alternatively-activated macrophages. Shown is a representative lesion area. The bar indicates 100 µm. **B:** Corresponding quantification of n=5 mice per experimental group. Data are presented as mean values -/+ SD. Statistical significance was determined using the t-test and statistical significance is indicated by a p value < 0.05. Source data are provided as a Source Data file.

Supplementary Figure S8

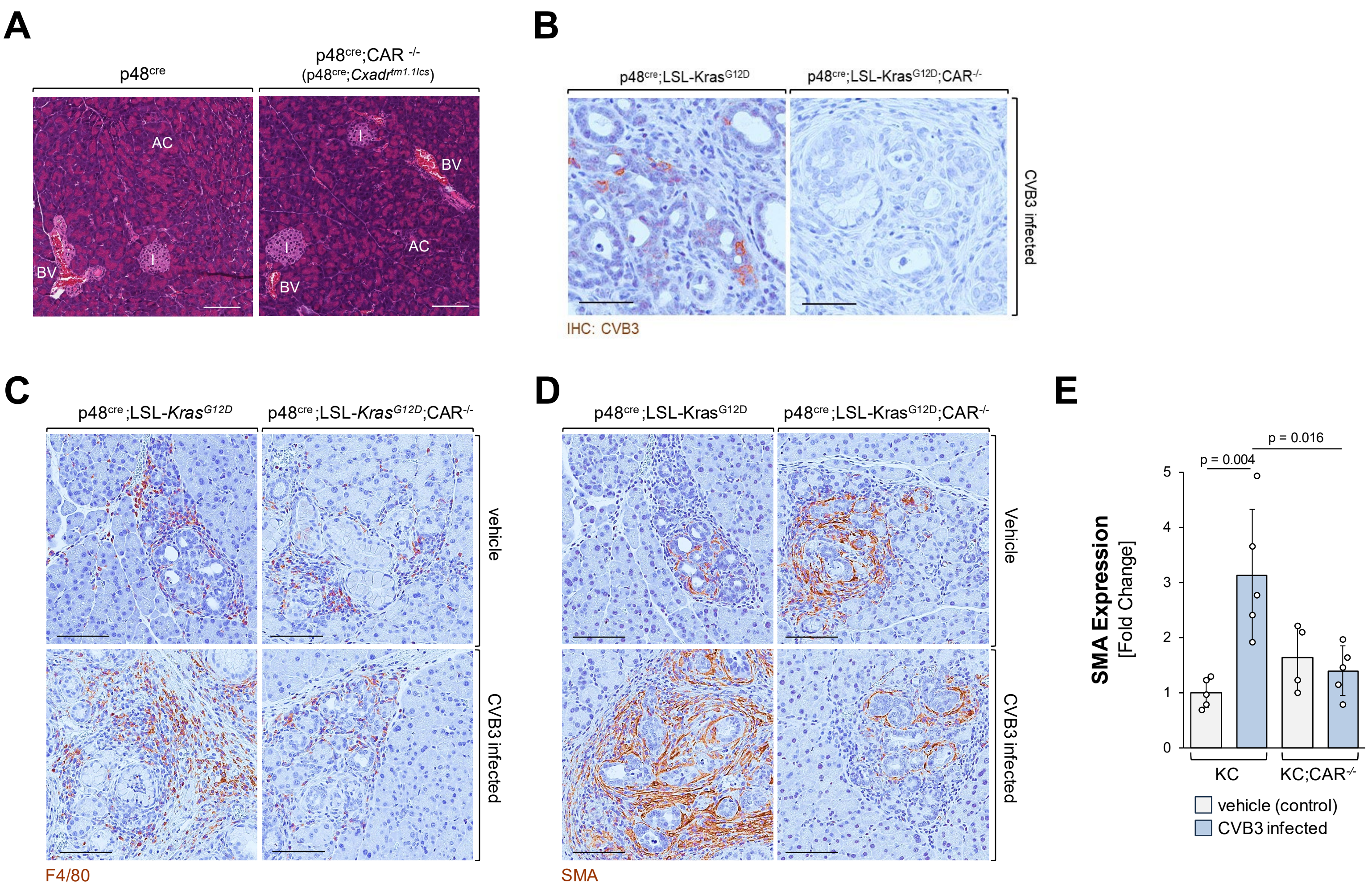

Supplementary Figure S8. A: An acinar cell specific knockout of CAR (p48^{cre};*Cxadr*$^{tm1.1lcs}$) has no phenotypic effect and does not affect normal pancreas morphology. Shown is a representative pancreas area of 16 weeks old mice. AC = normal acinar cell area, I = islet, BV = blood vessel. The scale bar represents 100 µm. **B: Control for Fig. 4.** Detection of CVB3 (IHC) in pancreata of KC or KC;CAR$^{-/-}$mice, 6 weeks after infection with CVB3. The scale bar represents 50 µm. Shown is a representative picture from n=5 mice per group that were analyzed. **C: Representative tissue areas for the analyses shown in Fig. 4C.** Pancreatic tissues of KC or KC;CAR$^{-/-}$ mice either vehicle treated or infected with CVB3 were analyzed by IHC for presence of macrophages (F4/80). The bar indicates 100 µm. **D, E: CVB3 infection increases the presence of SMA+ fibroblasts and this is blocked when CAR is knocked out. D:** Pancreatic tissues of KC or KC;CAR$^{-/-}$ mice either vehicle treated or infected with CVB3 were analyzed by IHC for presence of SMA+ cells. The bar indicates 100 µm. **E:** Corresponding quantification of n=5 mice per experimental group. Data are presented as mean values -/+ SD. Source data are provided as a Source Data file.

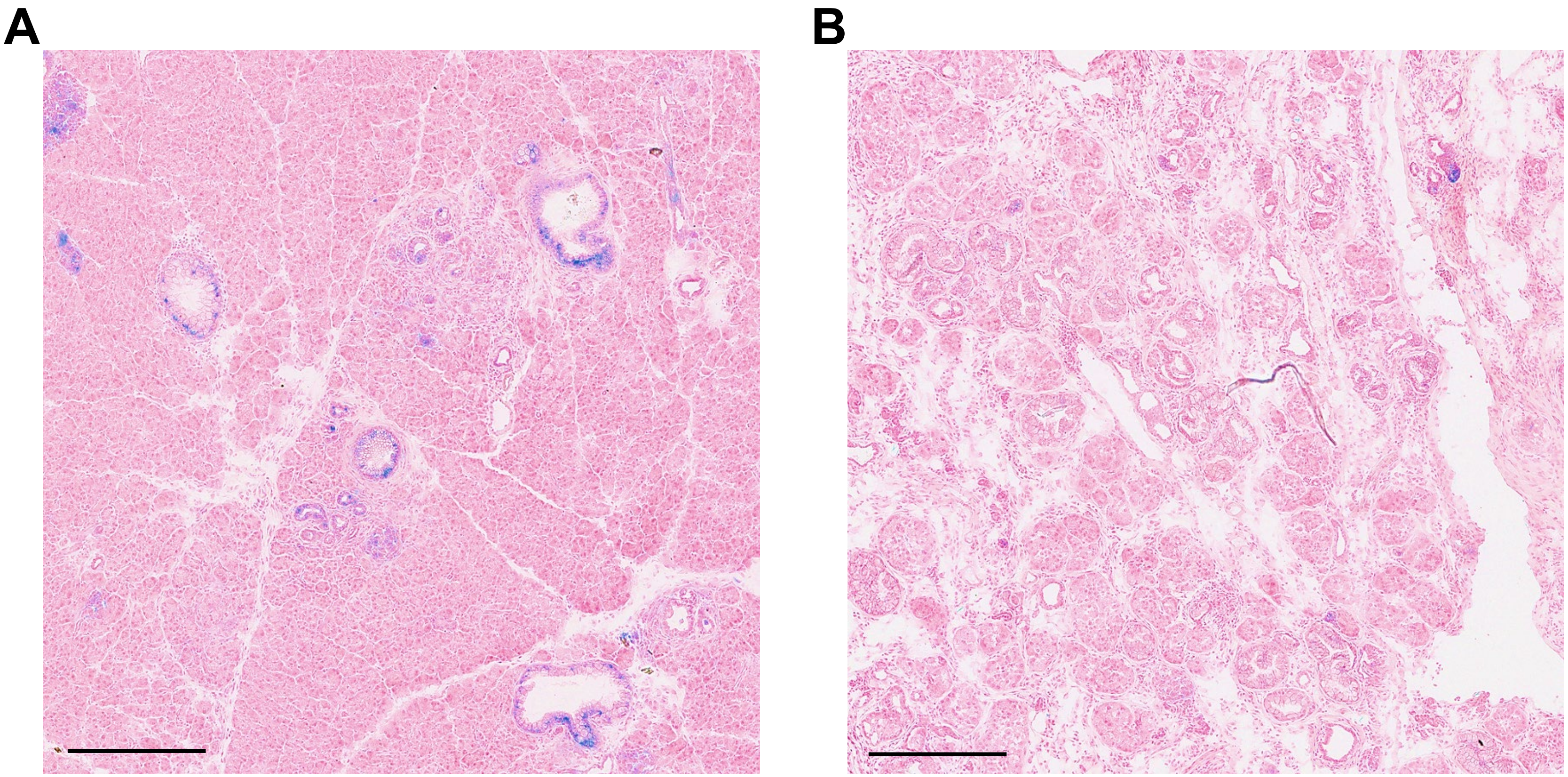

Supplementary Figure S9. Supplement to Figure 5A. **A, B:** Shown is a representative, larger overview area of the pancreata of KC mice either control treated (**A**) or infected with CVB3 (**B**) from Figure 5A. Samples were stained with the Senescence β-Gal staining kit from Cell Signaling and then counterstained with Nuclear Fast Red. The bar indicates 300 µm.

Supplementary Figure S10

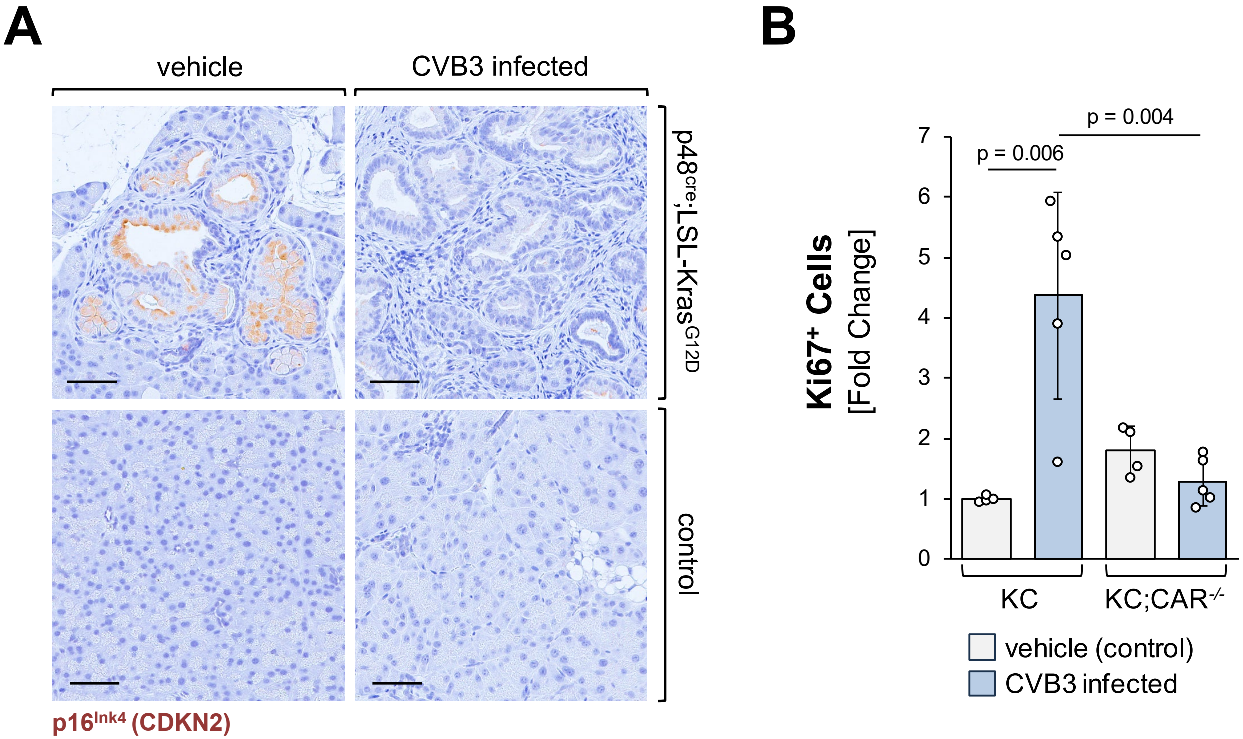

Supplementary Figure S10. A: Representative tissue areas for the analyses shown in Fig. 5B. Pancreatic tissues of KC or control mice either vehicle treated or infected with CVB3 (n=3 per experimental group) were analyzed by IHC for presence of $p16^{Ink4}$ (CDKN2). The bar indicates 50 µm. **B: Analyses corresponding to the representative tissue areas shown in Fig. 5C.** Pancreatic tissues of KC or KC;$CAR^{-/-}$ mice either vehicle treated or infected with CVB3 were stained proliferating cells (IHC for Ki67). Shown is a quantification n=5 mice per experimental group. Data are presented as mean values -/+ SD. Source data are provided as a Source Data file.

Supplementary Table 1

Antibody	Source	Catalog Number	IHC	PLA
CAR	Abcam	ab189216	1:1000	1:100
CD3	Abcam	ab5690	1:400	
CD4	Abcam	ab183685	1:1000	
CD8	Abcam	ab209775	1:500	
CK-19	Santa Cruz	SC-33111	1:100	
COX-2	Cayman Chemical	160126	1:800	
CVB1	Millipore	MAB944	1:500	
CVB2	Millipore	MAB946	1:100	
CVB3	Millipore	MAB948	1:400	1:400
CVB4	Millipore	MAB941	1:1000	
CVB5	Millipore	MAB943	1:200	
CVB6	Millipore	MAB945	1:500	
DCLK1	Abcam	ab37994	1:200	
F4/80	AbD Serotec	MCA497R	1:200	
Ki67	Abcam	ab15580	1:800	
Ly6B.2	AbD Serotec	MCA771G	1:2000	
$p16^{Ink4}$	Abcam	ab189034	1:200	
SMA	Abcam	ab5694	1:200	
Ym1	STEMCELL Technologies	60130	1:200	

Supplementary Table 1. **Antibodies and dilutions.** Antibodies used were from the following sources: Abcam (Cambridge, MA), AbD Serotech (Raleigh, NC), Millipore (Billerica, MA), Santa Cruz Biotechnology (Dallas, TX), Cayman Chemical (Ann Arbor, MI), STEMCELL Technologies (Vancouver, Canada).
