# Supplementary material for: Coxsackievirus and adenovirus receptor expression facilitates enteroviral infections to drive the development of pancreatic cancer
Source: Nat Commun. 2024 Dec 4;15:10547. doi: 10.1038/s41467-024-55043-x (PMC11615305; doi:10.1038/s41467-024-55043-x)
Supplement: Supplementary file 2 — Reporting Summary [file 41467_2024_55043_MOESM2_ESM.pdf]

Corresponding author(s): Peter Storz

Last updated by author(s): 11-11-2024

Reporting Summary

Nature Portfolio wishes to improve the reproducibility of the work that we publish. This form provides structure for consistency and transparency in reporting. For further information on Nature Portfolio policies, see our Editorial Policies and the Editorial Policy Checklist.

Statistics

For all statistical analyses, confirm that the following items are present in the figure legend, table legend, main text, or Methods section.

n/a	Confirmed	
☐	☒	The exact sample size (*n*) for each experimental group/condition, given as a discrete number and unit of measurement
☐	☒	A statement on whether measurements were taken from distinct samples or whether the same sample was measured repeatedly
☐	☒	The statistical test(s) used AND whether they are one- or two-sided<br>*Only common tests should be described solely by name; describe more complex techniques in the Methods section.*
☒	☐	A description of all covariates tested
☒	☐	A description of any assumptions or corrections, such as tests of normality and adjustment for multiple comparisons
☐	☒	A full description of the statistical parameters including central tendency (e.g. means) or other basic estimates (e.g. regression coefficient) AND variation (e.g. standard deviation) or associated estimates of uncertainty (e.g. confidence intervals)
☐	☒	For null hypothesis testing, the test statistic (e.g. *F*, *t*, *r*) with confidence intervals, effect sizes, degrees of freedom and *P* value noted<br>*Give P values as exact values whenever suitable.*
☒	☐	For Bayesian analysis, information on the choice of priors and Markov chain Monte Carlo settings
☒	☐	For hierarchical and complex designs, identification of the appropriate level for tests and full reporting of outcomes
☒	☐	Estimates of effect sizes (e.g. Cohen's *d*, Pearson's *r*), indicating how they were calculated

Our web collection on statistics for biologists contains articles on many of the points above.

Software and code

Policy information about availability of computer code

Data collection	Agilent 2100 Bioanalyzer (Agilent, Santa Clara, CA) - RNAseq data acquisition Fig. 1A; BIO-RAD Molecule Imager ChemiDoc XRS+ Imaging System - Supplemental Fig. 3B; Leica Aperio AT2 ScanScope slide scanner (Leica Biosystems, Buffalo Grove, IL) and ImageScope (v12.4.6.7001) software (Aperio, Vista, CA) - for all image acquisition and tissue pictures; QuantStudio 7 Flex Real-Time PCR System (Applied Biosystems) for quantitative PCR.
Data analysis	Adobe Photoshop 2025 (26.0.0) – all tissue pictures, Supplemental Fig. 3B; Adobe Illustrator 2025 (29.0.1) – final figures; MS Excel (Bar Graphs); MS PowerPoint (Schemes, Pie Graphs, Supplemental Figures); GraphPad Prism 9.2.0 – For Figures 1A, 1D, 6B; for all statistical analyses.

For manuscripts utilizing custom algorithms or software that are central to the research but not yet described in published literature, software must be made available to editors and reviewers. We strongly encourage code deposition in a community repository (e.g. GitHub). See the Nature Portfolio guidelines for submitting code & software for further information.

Data

Policy information about availability of data

All manuscripts must include a data availability statement. This statement should provide the following information, where applicable:

- Accession codes, unique identifiers, or web links for publicly available datasets
- A description of any restrictions on data availability
- For clinical datasets or third party data, please ensure that the statement adheres to our policy

A data availability statement has been included in the manuscript. Source data are provided with this paper. The Gene Expression Omnibus (GEO) accession code for data shown in Fig. 1A is GSE280352. A link is provided in the manuscript.

Research involving human participants, their data, or biological material

Policy information about studies with human participants or human data. See also policy information about sex, gender (identity/presentation), and sexual orientation and race, ethnicity and racism.

Reporting on sex and gender	Patient samples were de-identified for this study. Sex or gender were not considered.
Reporting on race, ethnicity, or other socially relevant groupings	All these factors were not considered.
Population characteristics	For the human tissue samples the only criteria were that they are positive for pancreatic ductal adenocarcinoma (PDA). All samples were de-identified and age and other patient-related information were not considered.
Recruitment	The TMAs with de-identified tissue samples were obtained from different sources as indicated in the manuscript.
Ethics oversight	This research according to NIH criteria is considered non-human research. IHC and ISH analyses of TMAs was approved by the Mayo Clinic Institutional Review Board (IRB) under protocol numbers listed in the manuscript.

Note that full information on the approval of the study protocol must also be provided in the manuscript.

Field-specific reporting

Please select the one below that is the best fit for your research. If you are not sure, read the appropriate sections before making your selection.

[x] Life sciences [ ] Behavioural & social sciences [ ] Ecological, evolutionary & environmental sciences

For a reference copy of the document with all sections, see nature.com/documents/nr-reporting-summary-flat.pdf

Life sciences study design

All studies must disclose on these points even when the disclosure is negative.

Sample size	Sample size or number of experimental animals was determined by power analysis such that it was sufficient to detect changes with 95% confidence between experimental conditions.
Data exclusions	No data were excluded form the analyses.
Replication	Every experiment shown was performed in 3 to 6 replicates. All replicates were reproducible. All quantification analyses were performed with 3-6 independent and individual samples.
Randomization	In animal studies, all mice (of both gender) were randomly assigned to the experimental groups.
Blinding	For all data analyses, such as for example the analyses of TMAs or IHC staining of mouse pancreata, investigators were blinded to group allocation during data collection and analysis (blinded study).

Reporting for specific materials, systems and methods

We require information from authors about some types of materials, experimental systems and methods used in many studies. Here, indicate whether each material, system or method listed is relevant to your study. If you are not sure if a list item applies to your research, read the appropriate section before selecting a response.

Materials & experimental systems

n/a	Involved in the study
☐	☒ Antibodies
☒	☐ Eukaryotic cell lines
☒	☐ Palaeontology and archaeology
☐	☒ Animals and other organisms
☒	☐ Clinical data
☒	☐ Dual use research of concern
☒	☐ Plants

Methods

n/a	Involved in the study
☒	☐ ChIP-seq
☒	☐ Flow cytometry
☒	☐ MRI-based neuroimaging

Antibodies

Antibodies used

CVB1 (Millipore ;MAB944, used 1:500); CVB2 (Millipore ;MAB946, used 1:100); CVB3 (Millipore; MAB948, used 1:400); CVB4 (Millipore; MAB941, used 1:1000); CVB5 (Millipore; MAB943, used 1:200); CVB6 (Millipore; MAB945, used 1:500); CAR (Abcam; ab189216, used 1:1000); CK-19 (Santa Cruz; SC-33111, used 1:100); COX-2 (Cayman Chemical; 160126, used 1:800); CD3 (Abcam; ab5690, 1:400); CD4 (Abcam; ab183685, 1:1000); CD8 (Abcam; ab209775, 1:500); p16INK4 (Abcam; ab189034, 1:200); DCLK1 (Abcam; ab37994, 1:200); F4/80 (AbD Serotec; MCA497R, 1:200); Ly6B2 (AbD Serotec; MCA771G, 1:2000); SMA (Abcam; ab5694, 1:200); Ki67 (Abcam; ab15580, 1:800); Ym1 (Stemcell Technologies; 60130, 1:200). This information has been included in Table 1 in Supplemental Data S11.

Validation

All antibodies used (i.e. CVB antibodies) are from reliable companies (i.e. Abcam, Millipore) known for rigorous testing of the specificity of their antibodies. The Millipore CVB antibodies (CVB1-CVB6) were validated by the company via immunofluorescence. We also controlled results obtained with CVB3 antibodies by in situ hybridization highlighting CVB3 expressing cells (see Fig. 2C). The CAR antibody from Abcam was verified by the company using human small intestine tissue, HepG2 and COLO cell extracts. CD4, CD8, CD3 antibodies from Abcam were verified by the company using mouse, human and rat thymus tissue lysates. CD4, CD8, and CD3 antibodies and other antibodies in this study, including, F4/80 (verified by the company with cultured bone marrow cells), Ym1 (verified by the company with mouse peritoneal macrophages), Ly6B.2 (tested by the company on thioglycollate stimulated peritoneal macrophages from C57BL/6 mice), COX-2 (tested by the company on COX-2 recombinant, raw 264.7 microsomes and mouse kidney tissue), aSMA (tested by company), CK-19 (verified by the company on SK-BR-3 cell lysate and on human kidney tissue), Ki67 (tested by company), p16INK4 (tested by company) or DCLK1 (tested by company on mouse heart, human brain tissue, human hepatocarcinoma tissue and mouse small intestine) are routinely used in our field, and have been used and published by us and others for similar analyses (Liou GY et al. Cell Rep 2017; 19(7):1322, PMCID:PMC5510483; Bastea LI et al. Cancer Res 2019; 79 (7):1535-1548, PMCID:PMC6445670; Liu X et al. Sci Rep 2019; 9(1):10656, PMCID:PMC6650496; Fleming Martinez AK et al. iScience 2020; 24(1):102019, PMCID:PMC7820128; Pandey V et al. eLife 2021; 10:e60646, PMCID: PMC8360647; Fleming Martinez AK et al. iScience 2022; 25(5):104327, PMCID:PMC9118688; Liou GY et al. iScience 2023; 26(6):106820, PMCID:PMC10212997.

Animals and other research organisms

Policy information about studies involving animals; ARRIVE guidelines recommended for reporting animal research, and Sex and Gender in Research

Laboratory animals

LSL-KrasG12D mice (obtained from the NCI Mouse Repository; MMHCC) were crossed with p48cre mice (a gift from Dr. Pinku Mukherjee, University of North Carolina) to generate bi-transgenic p48cre;LSL-KrasG12D (KC) mice, C57BL/6J background. Cxadrtm1.1lcs/J mice (strain #017359, B6;129S2 background) were obtained from The Jackson Laboratory (Bar Harbor, ME). LSL-p53R172H and Pdx1cre/+ mice (both 129/SvJae/C57Bl/6 background) were a gift from Dr. Howard Crawford (Henry Ford Health). The mice were housed and bred in ventilated cages in a temperature and humidity-controlled barrier facility at Mayo Clinic, under a 12-hour dark/light cycle. Food and water were provided ad libitum. Mice were used at the age indicated in the figure legends. For the CVB3 infection studies KC (p48-cre; LSL-KrasG12D) and KC; CAR-/- mice were used at an age of 8 weeks.

Wild animals

N/A

Reporting on sex

Since initiation of pancreatic cancer in our models does not show gender specificity, mice of different sex were randomly enrolled in the study. However, sex is reported for experiments shown in Supplemental Data S4, the supplement to Fig. 3D, as well as in Figures 4B and 4C, and 6B to rule out that sex has obvious effects on CVB3 infection in the KC model.

Field-collected samples

N/A

Ethics oversight

All animal experiments were approved by the Mayo Clinic Institutional Animal Care and Use Committee (IACUC) and were performed in accordance with relevant institutional and national guidelines and regulations. Experiments were conducted under Mayo Clinic IACUC protocols A00001701-16-R19, A00006044-21-R24, A43615-15, A00003891-18-R24.

Note that full information on the approval of the study protocol must also be provided in the manuscript.

Plants

Seed stocks	N/A
Novel plant genotypes	N/A
Authentication	N/A
